# Effectiveness and perceptions of using templates in long-term condition reviews: a systematic synthesis of quantitative and qualitative studies

**DOI:** 10.3399/BJGP.2020.0963

**Published:** 2021-07-27

**Authors:** Mary Morrissey, Elizabeth Shepherd, Emma Kinley, Kirstie McClatchey, Hilary Pinnock

**Affiliations:** Health Protection Team, Public Health NHS Lothian, Edinburgh, UK.; Sioux Lookout First Nations Health Authority, Sioux Lookout, Canada.; Usher Institute of Population Health Sciences and Informatics, University of Edinburgh, Edinburgh, UK.; Usher Institute of Population Health Sciences and Informatics, University of Edinburgh, Edinburgh, UK.; Usher Institute of Population Health Sciences and Informatics, University of Edinburgh, Edinburgh, UK.

**Keywords:** chronic conditions, delivery of health care, long-term conditions, patient-centred care, primary care, review templates

## Abstract

**Background:**

Review templates are commonly used in long-term condition (LTC) consultations to standardise care for patients and promote consistent data recording. However, templates may affect interactions during the review and, potentially, inhibit patient-centred care.

**Aim:**

To systematically review the literature about the impact that LTC review templates have on process and health outcomes, and the views of health professionals and patients on using review templates in consultations.

**Design and setting:**

Parallel qualitative and quantitative systematic reviews.

**Method:**

Following Cochrane methodology, nine databases were searched (1995–2019; updated July 2020) for clinical trials and qualitative studies of LTC templates in healthcare settings. Duplicate selection, risk-of-bias assessment, and data extraction were performed. The quantitative and qualitative analyses were conducted in parallel, and findings synthesised narratively.

**Results:**

In total, 12 qualitative and 14 quantitative studies were included (two studies reported both qualitative and quantitative data, and were included in both analyses). Review templates were well used, but the only study to assess health outcomes showed no effect. Templates can improve documentation of key measures and act as a reminder tool; however, they can restrict the review process, and risk health professionals’ agendas being prioritised over those of patients. Templates may also limit opportunities to discuss individuals’ concerns about living with their condition and act as a barrier to providing patient-centred care.

**Conclusion:**

Future research should evaluate health, as well as process, outcomes. The potential benefits of templates in improving documentation should be balanced against concerns that ‘tick boxes’ may override patient agendas, unless templates are designed to promote patient-centred care.

## INTRODUCTION

Long-term conditions (LTCs) account for >15 million premature deaths each year globally,^1^ emphasising a need to invest in strategies to improve management. Contemporary healthcare for LTCs is founded on evidence-based interventions summarised in clinical guidelines that recommend management strategies to optimise outcomes and prevent complications.^2^ In contrast, the role of supported self-management and patient activation is also emphasised,^3^^–^4 and there is evidence that a patient-centred approach is associated with improved health outcomes.^5^^–^7 LTC management should seek to bridge these two concepts by promoting health professional adherence to recommended tasks while, simultaneously, addressing the patient’s needs and supporting self-management.^8^

Electronic disease templates are commonly used in healthcare systems^6^ to optimise, structure, and standardise evidence-based care for patients, and promote consistent data recording.^6^^,^^9^ However, concerns have been expressed that review templates encourage a checklist approach to consultations, thereby restricting communication and reducing opportunities for discussion about self-management.^10^^–^11 Templates have also been criticised as prioritising the data needs of the institution over those of individual patients.^6^

In the context of a National Institute for Health Research (NIHR)-funded programme of work to develop a strategy for implementing supported asthma self-management in primary care (IMP^2^ART: IMPlementing IMProved Asthma self-management as RouTine), the authors sought to understand existing qualitative and quantitative evidence related to the design of LTC templates. Specifically, they aimed to:

- investigate the effectiveness of review templates in LTC consultations in terms of improving process and health outcomes; and
- explore health professional and patient experiences of using review templates in consultations.

## METHOD

### Design

The parallel qualitative and quantitative systematic reviews (each undertaken by one researcher) followed Cochrane methodology,^12^ and are reported according to Preferred Reporting Items for Systematic reviews and Meta-Analyses (PRISMA) standards.^13^ All aspects of the reviews’ design (searches, inclusion/exclusion criteria, outcomes, analysis) were specified a priori in two protocols.

**Table:** How this fits in

Electronic disease templates are commonly used in healthcare systems to optimise and standardise evidence-based care for patients during long-term condition (LTC) reviews. However, there are concerns that review templates can be a ‘tick-box’ exercise that has a negative impact on patient-centred care. Findings from qualitative and quantitative studies exploring the utility and impact of templates in LTC care were synthesised; the findings highlight the need to improve template design, with particular focus on supporting self-management and patient centredness.

### Search strategy

Qualitative and quantitative searches were performed independently in June 2019 using the following databases: MEDLINE (Ovid), EMBASE (Ovid), Web of Science, CINAHL (EBSCOhost), PsycINFO (Ovid), and British Nursing Index (ProQuest). Additionally, ASSIA and Sociological Abstracts (via ProQuest) were also searched for qualitative studies, and the Cochrane Central Register of Controlled Trials (CENTRAL) database was searched for quantitative studies. Reference lists were hand-searched and forward citation tracking of included studies was completed. Searches commenced for studies published from 1995, when the increasing importance of guidelines and advances in technology led to the widespread adoption of computerised medical records,^14^ which facilitated the use of templates and the secondary use of data.^14^ Prior to submission for publication (July 2020), forward citation tracking was carried out on all included studies, which is recognised as an efficient approach to updating reviews.^15^

### Definition of templates

Templates were defined as forms (paper or electronic), checklists, questionnaires, proformas, or smart forms that aim to:

- support structured management of patients;
- promote a systematic approach to care delivery;
- enable data recording, data sharing, and information retrieval;
- assure high-quality care delivery in line with evidence-based guidelines; and/or
- produce aggregated data that can be used to assess institution performance.

### Search terms

Databases were searched using terms to identify studies about review templates and LTCs. In addition, filters relating to methods were included, for example, ‘qualitative’ and ‘randomised controlled trial’. Full search terms and strategies for the qualitative and quantitative searches are given in Supplementary Table S1.

### Eligibility criteria

Table 1 displays the eligibility criteria for both searches guided by the Population, phenomena of Interest, and Context (PICo) and Population/Patient, Intervention, Comparison, Outcome, and Study design (PICOS) frameworks. Studies not published in the English language were excluded.

**Table 1.** Inclusion and exclusion criteria

	**Inclusion criteria**	**Exclusion criteria**
**Qualitative search (PICo framework)**		
Population	Patients with LTCs and health professionals managing patients with LTCs	Includes patients with no LTCs and health professionals’ management of patients with no LTCs
Phenomena of interest	Use of review templates (electronic or paper) in the clinical management of LTCs	Computerised decision-support systems, other reminders or record systems
Context	Views and experiences of review templates for clinical management of LTCs, from the patient or health professional perspective	Views and experiences that are not related to review templates used for clinical management of LTCs in a healthcare setting, from the patient or health professional perspective

**Quantitative search (PICOS framework)**		

Population	Health professionals working in LTC care	Non-health professionals, non-LTC review consultations
Intervention	Electronic or paper review templates meeting reviewers’ stated definition	Computerised decision-support systems, other reminders or record systems
Comparison Outcome	Standardised/regular LTC care not using templates Primary: process (comprehensiveness, compliance with guidelines, frequency of use). Secondary: patient health outcomes (unscheduled care, symptom control)	Studies not conducted in a healthcare setting No report on any outcomes of interest
Study design	Randomised controlled trials; quasi-experimental, non-randomised; mixed method	Study designs that did not meet the inclusion criteria

*LTC = long-term condition. PICo = Population, phenomena of Interest, and Context. PICOS = Population/Patient, Intervention, Comparison, Outcome, Study design.*

### Study selection

After de-duplication using Covidence (https://www.covidence.org), titles/abstracts were screened by two reviewers for the qualitative review and three reviewers for the quantitative review; potentially eligible full texts were then screened against the review criteria by three reviewers. Disagreements at any stage in the screening process were resolved through team discussion.

### Data extraction and quality assessment

Data were extracted by one reviewer for each of the qualitative and quantitative reviews, and independently checked by another reviewer. Qualitative studies were quality assessed by one reviewer, using the Critical Appraisal Skills Programme (CASP) checklist^16^ for qualitative research. Quantitative studies were assessed for risk of bias by a different reviewer. Randomised controlled trials (RCTs) were assessed using the Cochrane risk-ofbias assessment tool,^17^ and non-randomised studies were assessed using the Risk Of Bias In Non-randomised Studies — of Interventions (ROBINS-I).^18^ All risk-of-bias and quality assessments were independently checked by one other reviewer.

### Data synthesis

Due to the high level of heterogeneity across studies, a narrative synthesis^19^ was conducted; data from the qualitative and quantitative studies were initially synthesised separately. An overarching synthesis and interpretation were developed with a multidisciplinary group comprising academics, primary care clinicians, and health psychologists.

## RESULTS

**Figure 1.**
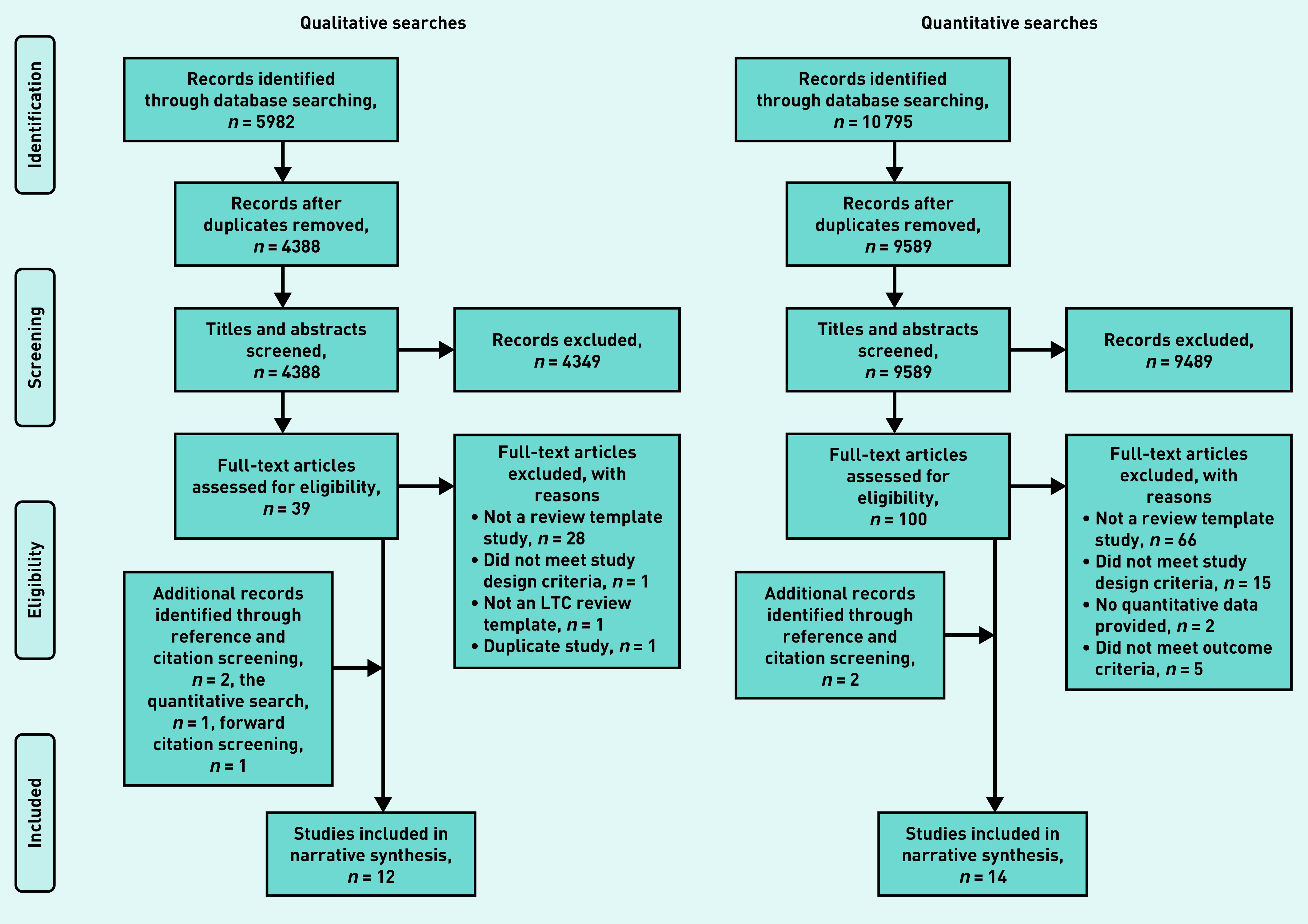
*Review process (PRISMA flow diagram): details of review process. PRISMA = Preferred Reporting Items for Systematic Reviews and Meta-Analyses.*

The qualitative search identified 12 studies,^6^^,^^9^^–^11^,^^20^^–^27 and the quantitative search identified 14 studies;^20^^–^21^,^^28^^–^39 the process is outlined in Figure 1). Two studies^20^^–^21 reported both qualitative and quantitative data and were included in both analyses. Characteristics and key interpretations of the included studies are shown in Supplementary Table S2 (qualitative) and Supplementary Table S3 (quantitative).

### Study characteristics

Qualitative studies were published between 1999 and 2019, and were undertaken in Australia (*n* = 1),^22^ South Africa (*n* = 1),^20^ and the UK (*n* = 10),^6^^,^^9^^–^11^,^^21^^,^^23^^–^27 in primary care practices and community health centres. The quantitative studies were published between 1999 and 2018, and were undertaken in Canada (*n* = 1);^28^ Kenya (*n* = 1);^29^ South Africa (*n* = 2);^20^^,^^30^ the UK (*n* = 2);^21^^,^^31^ the US (*n* = 8),^32^^–^39 in primary care practices, paediatric hospitals, community health centres, ambulatory care clinics, and mobile clinics. Multiple long-term conditions were included in the studies, commonly asthma, diabetes, and hypertension. Of the 24 unique studies, nine evaluated existing templates already in use in clinical practice,^9^^–^11^,^^22^^–^23^,^^25^^–^27^,^^31^ eight studies developed templates in a programme of research with the primary intention of embedding in routine practice,^20^^,^^24^^,^^28^^,^^30^^,^^32^^,^^37^^–^39 and seven studies had developed templates for research purposes that were subsequently embedded in clinical practice.^6^^,^^21^^,^^29^^,^^33^^–^36 Detailed study characteristics can be found for the qualitative and quantitative studies in Supplementary Table S2 and Supplementary Table S3 respectively.

### Quality and risk of bias

The qualitative quality assessment found that all but one study scored greater than seven out of a possible 10 on the CASP checklist.^22^ The quantitative risk-of-bias assessment found that all four RCTs had some concerns,^20^^–^21^,^^33^^,^^34^ and all of the non-randomised studies had a moderate-to-serious risk of bias.^28^^–^32^,^^35^^–^39 Full assessment details are detailed in Supplementary Table S4 for the qualitative studies, and Supplementary Figure S1 and Supplementary Figure S2 for the quantitative studies.

### Qualitative synthesis

#### Template design and data collection

Health professionals found templates acted as a reminder tool during consultations.^6^^,^^10^^,^^20^^–^24 As one nurse reported:

*‘I think they’re absolutely spot on, the templates. They’re just like reminders to make sure you don’t miss anything and they just make life a lot easier, basically*. *’*^23^

Templates established structure and made priorities clear, resulting in more efficient reviews.^20^^,^^23^^,^^25^^–^26 Conversely, rigid template design could be restrictive^11^^,^^21^ if structure was followed so closely that questions appeared out of context.^25^ Furthermore, over-reliance on structure reduced the health professionals’ opportunities to use their own medical knowledge and skills.^27^ Although some nurses expressed that templates *‘make life a lot easier’*,^23^ they also commented that templates mean *‘you don’t really have to think a lot for yourself*. *’* 10 Templates were viewed as inflexible if they did not provide space to record important additional comments.^20^^–^21 Additionally, a ‘tick-box’ design, as opposed to free-text comments, forced health professionals to categorise patients’ status, overriding nuances.^11^^,^^23^

One GP stated:

*‘I don’t want a load of prompts and a load of forms to fill in and click and buttons.’*^6^ [GP]

#### Competing agendas

Templates encouraged health professionals to prioritise their agenda over that of the patients.^10^^,^^11^^,^^25^ Patients had to work hard to integrate their own concerns into discussions and, even when successful, health professionals used the template to steer patients back to tasks.^11^ One template, the first question on which enquired about the patient’s agenda — namely, *‘What is the most important health problem that you would like us to work on over the next few months?’* — was valued by health professionals and patients.^6^

In some contexts, completing templates was an essential task, as it was how a practice secured its income;^9^ as such, health professionals felt under pressure to complete tasks and ‘tick the boxes’ that were related to evidence-based quality indicators.^10^^,^^23^^,^^26^ One nurse stated:

*‘That becomes number crunching, ticking boxes and that’s the bit I don’t like*. *’*^23^

#### Shaping patient–practitioner interactions

Template use could reduce eye contact and disrupt dialogue.^6^ When patients ‘digressed’ from the template tasks to talk about their concerns, some nurses used a shift in gaze towards the computer template to disturb the patients’ narrative and turn the conversation back to the next task.^25^^,^^27^ Templates caused less disjunction when screen positioning did not require clinicians to turn away from the patient.^6^ Nurses also used body positioning to indicate that the template had their full attention, by turning their whole body towards the screen, signalling lack of interest, and limiting the patients’ narrative.^25^^,^^27^ More positively, patients became familiar with the health professionals’ priorities imposed by the template and knew what to expect of the review process and understood what was deemed acceptable during the review.^25^

GPs interviewed felt that:

*‘*[templates were too] *business focused and took away from real doctoring.’*^22^ [GP]

#### Impact on patient centred-care

Health professionals acknowledged that template use could turn reviews into a tick-box exercise, which inhibited patient-centred care,^10^^,^^23^^–^24^,^^26^ with review appointments becoming focused on collecting data rather than being an opportunity for patients to discuss treatment options for managing their condition.^23^ As one nurse commented:

*‘You spend more time looking at the screen and ticking boxes than actually looking at the person who’s come to see you, which is not very nice for the patient.’*^26^

There was a risk of health professionals avoiding discussing patients’ concerns if they were not related to the condition under review,^11^^,^^25^ with patients expressing dissatisfaction if their problems were not addressed.^9^ One patient noted that:

*‘This gives me that kind of overview where you think “well, I’m the person that’s getting attended here, it’s not what this GP wants or thinks it’s what … my needs are”.’*^6^

Conversely, patients who were asked about their concerns responded positively and felt heard.^6^

GPs suggested that templates could be improved by enabling them to cater for patients with multiple conditions.^22^ Some health professionals adapted their templates and practice to facilitate patient-centred care — for example, by extending appointment times, adding free-text comment boxes, employing strategies to involve patients in the review, or by hand writing notes and completing the template when the patient had left.^9^^,^^11^^,^^21^^–^23

#### Template impact on treatment options, self-management, and health promotion

Some health professionals considered that templates encouraged a pharmacological approach to management, despite patients often preferring non-pharmacological options.^10^^,^^23^ Using the template, GPs shifted topics away from the patient-initiated self-management topics — for example, reducing medication need — to a discussion of options around the need for medication,^10^ which might deter patients from attending reviews.^23^

Health professionals felt that following the template and raising multiple health-promotion topics — for example, smoking, diet, alcohol — could cause upset and lead to the patient feeling criticised.^10^ One nurse noted an occasion when this had happened:

*‘I mean she was feeling a bit sort of got at, the fact that I’d already had the diet and the alcohol. And then smoking was the last straw really*.*’*^10^

As a result, nurses tended to avoid these lifestyle topics to preserve the patient relationship.^10^ Conversely, however, some nurses used the template as an excuse for asking self-management questions.^10^

#### Health professional differences in template use

Nurses, and staff with less training such as healthcare assistants, felt constrained to ‘obey’ templates, whereas GPs were happier to override template requirements.^23^ GPs often considered templates as too detailed, whereas nurses felt the detail was necessary.^21^ GPs who were provided with a short template were more able to integrate it into their consultations than nurses using relatively long templates in LTC reviews,^6^ although they did not always explore the patient’s agenda if they lacked the required expertise.^6^

Although nurses engaged conversationally with patients’ social circumstances, most GPs referred to biopsychosocial circumstances as context for patients’ health.^6^ Staff with less training, such as healthcare assistants, felt less equipped.^23^

It was noted that nurses initiated self-management dialogue more frequently than GPs.^10^

Some example quotes illustrating nurse and GP approaches to template use were:

*‘Yeah, you’ve got an agenda. They* [the patient] *may well have an agenda. And I tend to, rightly or wrongly, get my agenda first. You know, make sure my agenda’s done.’*^10^

[Nurse]

*‘There will be another agenda I’ll be running side by side … I’ve been able to cope OK with that.’*^23^

[GP]

### Quantitative synthesis

#### Use of templates

Overall, the majority of studies reported a rapid uptake or increase in the use of templates over the study period;^21^^,^^29^^–^32^,^^35^^–^36 however, one study (in which some concerns about risk of bias were highlighted) reported that <60% of patients’ folders contained the template being studied.^20^

#### Impact on documentation

Of the 14 included studies, 11 (all at moderate risk of bias or with some concerns) reported that review templates statistically significantly improved the documentation of key measures for their respective LTC.^28^^–^30^,^^32^^–^39 The studies that did not improve documentation reported lack of engagement with the research process and excessive workload undermining the ability to complete the template.^20^^–^21

Across the included studies, templates were reported to have the greatest effect on the process of disease management, including improved documentation of unscheduled care^32^^,^^37^^,^^39^ and symptoms.^34^ Templates were associated with a statistically significant improvement in the recording of condition severity’,^29^^,^^32^^,^^35^^,^^37^^,^^39^ with a change in documentation between 20% (*P* = 0.0013)^35^ and 73% (*P*<0.001).^39^ Statistically significant improvement in documentation was also noted for environmental exposure (for example, mould, occupational hazards).^29^^,^^32^

One asthma template study reported a statistically significant increase in the documentation of changes in care plans, including social work referral, subspecialty consultation, and medicine change (from 49% to 63%, *P* = 0.0006).^32^

The results were mixed with regards to complications or comorbidities, and changes in care plans that were documented. With regard to asthma, for example, documentation of an asthma action plan provision was mixed: one study found that documentation increased (from 10% to 74%, *P* = 0.001),^37^ whereas one found no statistically significant difference.^34^

Specifically, the impact on the documentation of prescribed controller medication was mixed, with some studies indicating a statistically significant improvement in documentation,^36^^,^^39^ while another study did not.^37^ One study observed increased documentation of inhaled corticosteroid use before and after template implementation (from 39.4% to 51.1%, *P* = 0.0170).^35^

Studies involving patients with hypertension and diabetes found mixed results in documentation changes regarding complications, with one study finding a statistically significant improvement in documentation,^29^ and another study finding no difference.^20^

The only study to report on family history (for example, history of smokers in family) did not report a statistically significant change in documentation.^34^

#### Impact on health outcomes

The only study to report on health outcomes (for which there was some concern about risk of bias) showed no statistically significant effect on glycaemic control for patients with diabetes or blood pressure control for those with hypertension following a template being introduced 1 year before the study was undertaken.^20^

## DISCUSSION

### Summary

In total, 24 unique studies investigating the use of templates in review consultations were identified. The overarching findings show that, even when review templates were well used, the limited evidence does not suggest that they improve patient-related outcomes. Health professionals perceived that templates were a helpful reminder tool during consultations, and the controlled trials confirmed that they could improve documentation of key measures in terms of adherence to guidelines. Templates were regarded positively in terms of structuring reviews and establishing clear priority tasks, but conversely were perceived as restricting the review process to ‘ticking boxes’, and risked prioritising the health professional’s agenda over that of the patient. In addition, it was found that templates may limit the opportunity to discuss self-management topics and act as a barrier to providing patient-centred care.

### Strengths and limitations

To the authors’ knowledge, this is the first systematic review to synthesise the effectiveness of review templates in LTC consultations with the views and experiences of health professionals and patients. The multidisciplinary team approach, which reduced the subjectivity of the qualitative synthesis and duplication of the selection processes used in this review, strengthen confidence in the findings. However, there are some limitations that should be noted.

Although broad search terms were used and 13 977 studies screened, some relevant studies may have been missed, specifically including studies not written in English (resources for translation were not available). Qualitative studies can be difficult to detect, but data saturation was reached with respect to the themes. All quantitative studies included in this review were assessed to be of moderate-to-serious risk of bias, thereby reducing the strength of the evidence presented. Further, the studies were heterogeneous, with varying LTCs and review templates, and initiated in diverse contexts, precluding meaningful meta-analysis.

### Comparison with existing literature

This review found that templates improved the documentation process, but with no evidence of improved health outcomes. This supports a prior review which showed that templates embedded in the electronic medical records have process benefits, but unclear improvements in clinical outcomes.^40^ It was also found that templates can act as a barrier to providing patient-centred care, which corroborates previous concerns that use of electronic health records negatively impacted on patient-centred communication.^41^

Patient-centred care is important for patients with LTCs,^42^ and evidence suggests that long-term patient outcomes may be improved when patients are involved in their treatment planning and supported to self-manage.^43^ This represents a tension in contemporary clinical practice, with policy rewarding both increased coding of guideline-recommended practice in pay-for-performance schemes,^44^ while simultaneously promoting personalised care.^42^^,^^45^ Increasing use of routine data and the ‘power of information’ is an additional driver for collecting process data,^46^ risking further marginalisation of the patient’s agenda. Template design could help — or hinder — the challenge of managing this tension.

### Implications for practice

There are a number of reasons why researchers, clinicians, and health service managers introduce templates into clinical practice. If improving processes or recording processes is the aim, then these findings suggest that templates have utility. In contrast, there was very little evidence about templates’ potential to improve clinical outcomes, although it should be noted that absence of evidence is not evidence of absence. Researchers developing or evaluating templates need to define their objective(s), consider the mechanism by which they believe a template can achieve that objective, and measure outcomes that demonstrate whether the objective has been achieved. In addition, the benefits need to be balanced against the perception that templates can reduce the focus on the patient during a consultation.

To improve patient-centredness, templates should open with a question that aims to establish the patient’s agenda,^6^ and should incorporate questions that ask patients about their main health concerns to allow initiation of discussion.^6^ Additionally, findings identified that patients feel dissatisfied with their unaddressed problems;^9^ a closing question on the template, to check whether the patient’s concerns were addressed in the consultation, may alleviate this.

It was found that templates were viewed as inflexible, and the studies included in this review suggested that they should incorporate open-text or flexible options that help balance patient and health professional agendas, and allow for documentation of patient concerns and multiple conditions.

Finally, existing evidence shows that supported self-management can reduce hospitalisations, accident and emergency attendances, and unscheduled consultations.^47^ As suggested by an included study,^10^ templates should incorporate more self-management questions and education to help health professionals encourage and educate patients in self-management practices. The IMP2ART programme of work, which is developing a strategy for implementing supported asthma self-management in primary care, will use these findings in the development of an asthma review template.

In conclusion, review templates were well used, although the limited evidence does not suggest that they improve patient-related outcomes. Templates can improve documentation of key measures, and act as a reminder tool during consultations; however, this can restrict the review process, and risks prioritising the health professional agenda over the patients’ concerns and act as a barrier to providing patient-centred care. Understanding and managing these potentially conflicting imperatives could lead to improved design of templates for use in LTC management.
